# Supplementary material for: AffyMAPSDetector: a software tool to characterize Affymetrix GeneChip™ expression arrays with respect to SNPs
Source: BMC Bioinformatics. 2007 Jul 30;8:276. doi: 10.1186/1471-2105-8-276 (PMC1959249; doi:10.1186/1471-2105-8-276)
Supplement: Additional file 13 — AffyMAPSDetector v1 source code. AffyMAPSDetector source code. [file 1471-2105-8-276-S13.zip › AffyMAPSDetector_v1_src/doc/index-files/index-17.html]

S-Index


|  |  |  |  |
| --- | --- | --- | --- |
| **Overview** \| Package \| Class \| Use \| **Tree** \| **Deprecated** \| **Index** \| **Help** | | |  |
| **PREV LETTER**   **NEXT LETTER** | **FRAMES**    **NO FRAMES**     **All Classes** |  |  |


A B C D E F G H I J L M N O P R S T V W X Y 

---


## **S**

**S** - Static variable in class affychip.snpdetector.util.PmMmHeatMapViewer: **SnpDetector** - class affychip.snpdetector.model.SnpDetector.: Title: Probe SNP Detector **SnpDetector()** - Constructor for class affychip.snpdetector.model.SnpDetector: **SnpFinder** - class affychip.snpdetector.model.SnpFinder.: Title: File Manager **SnpFinder()** - Constructor for class affychip.snpdetector.model.SnpFinder: **SnpInfo** - class affychip.snpdetector.model.SnpInfo.: Title: Probe SNP Detector **SnpInfo(String)** - Constructor for class affychip.snpdetector.model.SnpInfo: **SnpMatcher** - class affychip.snpdetector.model.SnpMatcher.: Title: Probe SNP Detector **SnpMatcher(DataState)** - Constructor for class affychip.snpdetector.model.SnpMatcher: **SnpMatcher.FileClosingShutdownHook** - class affychip.snpdetector.model.SnpMatcher.FileClosingShutdownHook.: Shudown hook that would ensure that if the program terminates abnormally then all opened data files, to which data is written, are properly closed without loosing any data that has not yet been commited to disk. **SnpMatcher.FileClosingShutdownHook(Writer[], Reader[])** - Constructor for class affychip.snpdetector.model.SnpMatcher.FileClosingShutdownHook: **SnpMatcher.FileClosingShutdownHook()** - Constructor for class affychip.snpdetector.model.SnpMatcher.FileClosingShutdownHook: **StatusEvent** - class affychip.snpdetector.model.event.StatusEvent.: Title: **StatusEvent(Object, String)** - Constructor for class affychip.snpdetector.model.event.StatusEvent: **StatusEvent(Object, String, Color)** - Constructor for class affychip.snpdetector.model.event.StatusEvent: **StatusEventListener** - interface affychip.snpdetector.model.event.StatusEventListener.: Title: **StepOnePanel** - class affychip.snpdetector.ui.StepOnePanel.: Title: Probe SNP Detector **StepOnePanel()** - Constructor for class affychip.snpdetector.ui.StepOnePanel: **StepThreePanel** - class affychip.snpdetector.ui.StepThreePanel.: Title: Probe SNP Detector **StepThreePanel()** - Constructor for class affychip.snpdetector.ui.StepThreePanel: **StepTwoPanel** - class affychip.snpdetector.ui.StepTwoPanel.: Title: Probe SNP Detector **StepTwoPanel()** - Constructor for class affychip.snpdetector.ui.StepTwoPanel: **scrollToCenter(JTable, int, int)** - Static method in class affychip.snpdetector.util.GuiUtils: **separators** - Static variable in class affychip.snpdetector.Constant: **setAlleles(String)** - Method in class affychip.snpdetector.model.SnpInfo: **setAttribute(Object, Object)** - Method in class jwf.WizardContext: Sets an attribute. **setAxisOrientation(String)** - Method in class affychip.snpdetector.model.SnpInfo: **setColor(double, Color)** - Method in class affychip.snpdetector.util.ColorGradient: **setDataState(HashMap)** - Method in class affychip.snpdetector.ui.AffyMapsDetectorFrame: This method wipes out any previous data state and regenrates it a fresh from the (name,value) pairs contained in the map. **setDescription(String)** - Method in class affychip.snpdetector.model.GeneInfo: **setFrameLocation(Window, double, double)** - Static method in class affychip.snpdetector.util.GuiUtils: Use this method if you want to place a `Window` relative to the top-left corner of graphical device screen. **setFunction(String)** - Method in class affychip.snpdetector.model.SnpInfo: **setGeneAnnot(HashMap)** - Method in class affychip.snpdetector.util.PmMmMatrixLoader: **setGeneIdentifierFieldIndex(int)** - Method in class affychip.snpdetector.model.DataState: **setGeneIdentifierFieldIndex(Integer)** - Method in class affychip.snpdetector.model.DataState: **setGeneIdentifierFieldIndex(String)** - Method in class affychip.snpdetector.model.DataState: **setGeneInfo(GeneInfo[])** - Method in class affychip.snpdetector.model.DataState: **setGeneInfo(GeneInfo)** - Method in class affychip.snpdetector.model.ProbeSet: **setGeneInfoFieldSeparator(String)** - Method in class affychip.snpdetector.model.DataState: **setGeneInfoFile(File)** - Method in class affychip.snpdetector.model.DataState: **setGeneInfoHeaders(String[])** - Method in class affychip.snpdetector.model.DataState: **setGeneInfoProbeSetIdFieldIndex(int)** - Method in class affychip.snpdetector.model.DataState: **setGeneInfoProbeSetIdFieldIndex(Integer)** - Method in class affychip.snpdetector.model.DataState: **setGeneInfoProbeSetIdFieldIndex(String)** - Method in class affychip.snpdetector.model.DataState: **setHaltExecution(boolean)** - Method in class affychip.snpdetector.model.SnpMatcher: **setHasSnpsFilename(String)** - Method in class affychip.snpdetector.model.DataState: **setHeteroZygosity(String)** - Method in class affychip.snpdetector.model.SnpInfo: **setI18NMap(Map)** - Method in class jwf.Wizard: **setLabelFont(JLabel)** - Static method in class jwf.WizardUtils: **setLocusLinkFieldIndex(int)** - Method in class affychip.snpdetector.model.DataState: **setLocusLinkFieldIndex(Integer)** - Method in class affychip.snpdetector.model.DataState: **setLocusLinkFieldIndex(String)** - Method in class affychip.snpdetector.model.DataState: **setMax(int)** - Method in class affychip.snpdetector.model.Probe: **setMemoryMeter()** - Method in class affychip.snpdetector.ui.AffyMapsDetectorUI: **setMenuItemsEnabled(boolean)** - Method in class affychip.snpdetector.ui.AffyMapsDetectorFrame: **setMiddlePosition(int)** - Method in class affychip.snpdetector.model.Probe: **setMin(int)** - Method in class affychip.snpdetector.model.Probe: **setMmPosFieldIndex(int)** - Method in class affychip.snpdetector.model.DataState: **setMmPosFieldIndex(Integer)** - Method in class affychip.snpdetector.model.DataState: **setMmPosFieldIndex(String)** - Method in class affychip.snpdetector.model.DataState: **setNoLocusLinkFilename(String)** - Method in class affychip.snpdetector.model.DataState: **setNoSnpsFilename(String)** - Method in class affychip.snpdetector.model.DataState: **setNucleotideAccNo(String)** - Method in class affychip.snpdetector.model.SnpInfo: **setOrganism(String)** - Method in class affychip.snpdetector.model.DataState: **setPmMmMatrixFile(String)** - Method in class affychip.snpdetector.util.PmMmHeatmapViewerModel: **setPmMmMatrixFile(File)** - Method in class affychip.snpdetector.util.PmMmHeatmapViewerModel: This method loads the PM/MM ratio matrix file and builds the data model for the heatmap viewer. **setPmMmMatrixFile(String)** - Method in class affychip.snpdetector.util.PmMmMatrixLoader: **setPmMmMatrixFile(File)** - Method in class affychip.snpdetector.util.PmMmMatrixLoader: This method loads the PM/MM ratio matrix file and builds the data model for the heatmap viewer. **setProbeInfoFieldSeparator(String)** - Method in class affychip.snpdetector.model.DataState: **setProbeInfoFile(File)** - Method in class affychip.snpdetector.model.DataState: **setProbeInfoHeaders(String[])** - Method in class affychip.snpdetector.model.DataState: **setProbeInfoProbeSetIdFieldIndex(int)** - Method in class affychip.snpdetector.model.DataState: **setProbeInfoProbeSetIdFieldIndex(Integer)** - Method in class affychip.snpdetector.model.DataState: **setProbeInfoProbeSetIdFieldIndex(String)** - Method in class affychip.snpdetector.model.DataState: **setProbeLocationIndicesFor(Probe)** - Method in class affychip.snpdetector.model.GeneInfo: This method finds starting index, ending index and index of 13th position of a given probe sequence relative to this gene's mRNA sequence. **setProbeSeqFieldIndex(int)** - Method in class affychip.snpdetector.model.DataState: **setProbeSeqFieldIndex(Integer)** - Method in class affychip.snpdetector.model.DataState: **setProbeSeqFieldIndex(String)** - Method in class affychip.snpdetector.model.DataState: **setProbeSet(HashMap)** - Method in class affychip.snpdetector.model.DataState: **setProtAccNo(String)** - Method in class affychip.snpdetector.model.SnpInfo: **setRefSeqAccNo(String)** - Method in class affychip.snpdetector.model.GeneInfo: **setRsClusterId(String)** - Method in class affychip.snpdetector.model.SnpInfo: **setSNPs(String[])** - Method in class affychip.snpdetector.model.Probe: **setSequence(String)** - Method in class affychip.snpdetector.model.GeneInfo: **setSnpClass(String)** - Method in class affychip.snpdetector.model.SnpInfo: **setSnpLoc(String)** - Method in class affychip.snpdetector.model.SnpInfo: **setStopExecution(boolean)** - Method in class affychip.snpdetector.model.SnpMatcher: **setTableData(Vector)** - Method in class affychip.snpdetector.ui.AffyMapsDetectorFrame: **setWebGeneInfoFilename(String)** - Method in class affychip.snpdetector.model.DataState: **setWebSnpInfoFilename(String)** - Method in class affychip.snpdetector.model.DataState: **setWizardContext(WizardContext)** - Method in class jwf.WizardPanel: Sets the context this wizard should use. **setXPosFieldIndex(int)** - Method in class affychip.snpdetector.model.DataState: **setXPosFieldIndex(Integer)** - Method in class affychip.snpdetector.model.DataState: **setXPosFieldIndex(String)** - Method in class affychip.snpdetector.model.DataState: **setYPosFieldIndex(int)** - Method in class affychip.snpdetector.model.DataState: **setYPosFieldIndex(Integer)** - Method in class affychip.snpdetector.model.DataState: **setYPosFieldIndex(String)** - Method in class affychip.snpdetector.model.DataState: **showErrorMessages(List)** - Method in class jwf.ErrorMessageBox: Show a list of messages and block until ok is pressed. **showFileChooser(Component)** - Static method in class jwf.WizardUtils: This method should be used to show if file system needs to be browsed. **start(WizardPanel)** - Method in class jwf.Wizard: Start this wizard with this panel. **startMatch()** - Method in class affychip.snpdetector.model.SnpMatcher: **statusUpdate(StatusEvent)** - Method in interface affychip.snpdetector.model.event.StatusEventListener: **statusUpdate(StatusEvent)** - Method in class affychip.snpdetector.ui.AffyMapsDetectorFrame

---


|  |  |  |  |
| --- | --- | --- | --- |
| **Overview** \| Package \| Class \| Use \| **Tree** \| **Deprecated** \| **Index** \| **Help** | | |  |
| **PREV LETTER**   **NEXT LETTER** | **FRAMES**    **NO FRAMES**     **All Classes** |  |  |


A B C D E F G H I J L M N O P R S T V W X Y 

---
